# Effectiveness of Primary Care Triple P on child psychosocial problems in preventive child healthcare: a randomized controlled trial

**DOI:** 10.1186/1741-7015-11-240

**Published:** 2013-11-11

**Authors:** Willem Spijkers, Daniëlle EMC Jansen, Sijmen A Reijneveld

**Affiliations:** 1 Department of Health Sciences, University Medical Center Groningen, University of Groningen, A. Deusinglaan 1, 9713 AV, Groningen, The Netherlands; 2 Department of Epidemiology, Municipal Health Service Groningen, Hanzeplein 120, 9713 GW, Groningen, The Netherlands; 3 Department of Sociology and Interuniversity Center for Social Science Theory and Methodology (ICS), University of Groningen, Grote Rozenstraat 31, 9712 TG, Groningen, The Netherlands

**Keywords:** Child behaviour disorders, Parenting, Early intervention, Randomized controlled trial

## Abstract

**Background:**

Psychosocial problems in children have adverse effects on the children, their families, and society, thus early intervention is important. Community pediatric services offer an ideal setting to detect problem behaviour in children and provide support to parents. The objective of this study was to assess the effectiveness of a Primary Care Triple P (PCTP) program compared with care as usual (UC) for parents of children with mild psychosocial problems after an initial, evidence-based screening in routine community pediatric care.

**Methods:**

We conducted a multicenter, randomized, controlled trial in community pediatric services in the Netherlands, enrolling parents of children with mild psychosocial problems. The population was identified by screening using the Strengths and Difficulties Questionnaire (SDQ) with a cut-off point of 11 or higher (that is, a subclinical score). We compared PCTP with UC, and measured the effects immediately after treatment and after 6 and 12 months. PCTP comprised four individual counseling sessions with the parent of 20 to 30 minutes each. The primary outcome measures were the child psychosocial problems as measured by the SDQ and the Eyberg Child Behaviour Inventory (ECBI).

**Results:**

In total, 81 families were recruited and randomized, and 67 provided post-intervention data. Both treatment groups improved after treatment, with the PCTP group improving only slightly more than the UC group on most measures. The maximum difference on the SDQ was 1.94 (95% CI = −0.30 to 4.19, *P* = 0.09) and 5.81 (95% CI = −3.37 to 14.99, *P* = 0.21) on the ECBI (n = 67). None of the differences between PCTP and UC was significant. In the subsidiary analyses, only one of the twenty outcomes (that is, SDQ conduct problems) was significant.

**Conclusions:**

PCTP did produce a reduction in psychosocial problems in children but had no statistically significant advantage over UC. In general, a few outcomes improved in both groups. Based on this admittedly underpowered study, we cannot conclude that PCTP is more effective than UC in preventive child healthcare.

**Trial registration:**

Nederlands Trial Register (Dutch Trial Register): NTR1338.

## Background

Psychosocial problems, such as aggressive behaviour, fear, and anxiety, are common in children and may lead to restrictions in daily functioning. According to population-based studies, 20% of Dutch children have some degree of psychosocial problems [1,2]. Psychosocial problems in children represent a considerable expense to society and are an important reason for using health care [3]. Moreover, these problems can have a large effect on a child’s future life [4]. Several studies have shown that psychosocial problem affect the child’s social competence, school performance, and later psychosocial development [5,6].

Parenting style and parental competencies are related to psychosocial problems in children, and are therefore valid determinants of the child problem behaviour to be targeted [7]. Dysfunctional parenting is more likely with parents who are uncertain about their parenting skills [8]. Research has shown that the earlier child behavioural problems commence, the greater the risk that they will become worse and persist in adulthood [9]. Early detection and treatment of these problems is therefore important, and may prevent difficult child behaviour occurring or worsening [10].

Community pediatric services offer an ideal setting for the early detection and treatment of child psychosocial problems [1,2]. In the Netherlands, such services offer preventive child healthcare (PCH) which is provided free of charge to all children. Early detection is already part of PCH in many countries, but effective tools for early treatment are still lacking. There is therefore a need for standardized parenting support interventions that are short term and suit the competences of child healthcare professionals (CHPs).

Interventions to address child psychosocial problems by enhancing parenting skills are becoming increasingly available, but evidence on their effectiveness in community pediatric services such as PCH is lacking. Primary Care Triple P (level 3 of the Triple P program [11,12]) may suit this purpose because it is short and matches the competences of the CHPs. PCTP aims to improve parenting skills in order to reduce child psychosocial problems. Although evidence is widely available on the effectiveness of the more intensive variants of Triple P, both in the Netherlands [13,14] and elsewhere [15-17], these studies have received some criticism [18-21]. Moreover, evidence on the effectiveness of PCTP is particularly scarce and inconclusive [16,18-21], and is lacking for the Netherlands. A randomized controlled trial (RCT) investigating the effects of PCTP has never been conducted before in the PCH system, to our knowledge. This study therefore aimed to determine the effectiveness of PCTP compared with usual care (UC) for parents of children with mild psychosocial problems.

## Methods

### Research design

We conducted an RCT with follow-up assessments after completion of the intervention, and 6 and 12 months later. A comprehensive description of the project objectives, diagnostic instruments, procedure, design, and analysis of this study is provided elsewhere [22]. In brief, prior to a routine PCH health examination, parents completed a screening questionnaire about psychosocial problems in children. Parents of children with sufficiently high scores for psychosocial problems were assigned at random to the experimental group (PCTP) or UC group unless the child had an existing formal psychiatric diagnosis or was currently receiving treatment for such problems.

The Medical Ethics Committee of the University Medical Center of Groningen approved the study protocol. We report the findings following the CONSORT guidelines [23].

### Participants

Participants were recruited from a normal-risk population of primary school children aged 9– to 11 years and their parents in four provinces in the Netherlands. The children were examined during a routine PCH screening. Recruitment started in September 2008 and ended in June 2011. If the children and the parents met the study inclusion criteria, the parents were approached to take part in the study. The intervention started within a month of inclusion in the study.

### Consent

Participation in this study was voluntary and all participants signed an informed consent form.

### Study inclusion and exclusion criteria

The inclusion criteria were: 1) child age 9 to 11 years; 2) total difficulties score for the child on the Strengths and Difficulties Questionnaire (SDQ) in the (sub-)clinical range (≥11). The exclusion criteria were: 1) child diagnosed with developmental delay, developmental disorder (for example, autism), conduct disorder, or attention deficit hyperactivity disorder; 2) child currently receiving treatment for behavioural problems; 3) child with a chronic disease involving three or more medical consultations in the previous 2 months; 4) parental divorce, death, or severe illness of someone to whom the child is attached (parent, sibling, grandparent, friend, nanny) in the previous 6 months; 5) parents in therapy for psychological or relationship problems; 6) parents unable to read or speak Dutch; 7) behavioural or emotional problems in the child beyond the scope of PCTP; 8) situations involving child safety such as child maltreatment, parental psychiatric disorder, or alcohol or drug abuse.

### Interventions

PCTP is a standardized, four-session intervention for children with mild to moderate psychosocial problems, and includes active skills training for parents. It combines advice, rehearsal, and self-evaluation to teach parents to manage one particular child behavioural problem, and involves four individual consultations of 20 to 30 minutes with the parents and their child. The intervention is described in greater detail elsewhere [24,25]. In the RCT, PCTP was delivered by eight Triple P practitioners (that is, PCH nurses) who were accredited for Triple P levels 2 and 3. An accredited Triple P trainer provided periodical supervision. Nurses who carried out PCTP were excluded from carrying out UC, and nurses who provided UC were not trained or otherwise acquainted with PCTP.

UC varied from advice to a home visit with no pre-specified guidelines. The lowest intensity of UC implied some advice and the highest intensity more intensive parenting support. In cases with an elevated SDQ total problem score (that is, ≥11), the CHP verified the severity of the situation with the parents and, sometimes, with schoolteachers. If the parents acknowledged that their child had psychosocial problems and that they themselves experienced parenting problems, the CHP tried to clarify the problem and provide parenting support. Generally, CHPs planned a maximum of three extra contact sessions with the parents.

### Measures

The primary outcome of the study was the presence of child psychosocial problems, and the secondary outcomes of the study related to parenting behaviour and parenting stress. The outcomes were assessed by means of questionnaires completed by the parents. Child psychosocial problems were measured by two instruments. The first was the SDQ [26,27], which consists of 25 items, divided into five subscales assessing pro-social behaviour, hyperactivity, emotional symptoms, conduct problems, and peer problems, and describing both positive and negative aspects of child behaviour. The SDQ Total Difficulties Score (SDQ-TDS, range 0 to 40) is the sum of the scores on all subscales except the pro-social behaviour subscale. The second was the Eyberg Child Behaviour Inventory (ECBI), which consists of 36 items on child behaviour (range 36 to 252 on the total scale), namely, oppositional defiant behaviour, inattentive behaviour, and conduct problems [28].

The secondary outcomes of the study were parenting behaviour and stress, because the intermediate goal of the intervention was to improve parenting. Parenting behaviour and parenting stress were measured by the: Parenting Scale (PS), consisting of 30 items on parental discipline, such as laxity, over-reaction, and verbosity (range 1 to 7 on the mean total scale) [29]; the Problem Setting and Behaviour Checklist (PSBC), which comprises 28 items on handling difficult day-to-day parenting situations (range: 1 to 10 on the mean total scale); the Parenting Stress Index (PSI; Dutch: Nijmeegse Ouderlijke Stress Index), short version, comprising 11 items on parenting-related depression and stress (range: 11 to 66 on the total scale) [30]; and the Depression Anxiety Stress Scale (DASS), covering 21 items on depression, anxiety, and stress symptoms (range: 0 to 126 on the total scale) [31].

The treatment integrity was measured by the number of intervention sessions per family delivered by the CHP. Furthermore, questions on family background such as marital status, parental educational level, parental work situation, household financial situation, family size, and age of the parents and the child were included.

### Sample size

In this study, 64 children were needed in each treatment arm (total 128) to show an effect size of 0.5 of PCTP regarding parent-reported child psychosocial problems (measured by the SDQ) with a power of 80% at *P* < 0.05.

### Randomization

A research assistant ensured that participants enrolled from PCH practice were eligible. Randomization was based on a computer-generated randomization program, with each child being randomized as they entered the study. To prevent unequal randomization, participants were pre-stratified and randomized by center using block randomization (blocks of six).

### Blinding

Participants were not informed about the type of the treatment they would receive, but due to the nature of the study, the nurses who delivered the interventions could not be blinded.

### Statistical methods

First, the baseline characteristics of the parents in the two treatment groups were compared, with χ^2^ tests for categorical variables and Student’s t-tests for continuous variables used to detect possible differences. All participants who completed the baseline measurement and at least one post-intervention measurement were included in the analyses. For the intention-to-treat (ITT) analysis, the final observation carried forward was used.

Second, we assessed the mean improvement between the baseline and the post-intervention measurements in both the intervention and in the UC group. We used multi-level regression analysis to assess the effect of the intervention compared with UC, in which each measurement per child was the first level, and the child the second level. In this way, we accounted for the dependency of the repeated measures within individuals and the variability between individuals. It should be noted that the resulting β values are not fully equal to the differences in improvements between the intervention and the UC groups, owing to the adjustments for consecutive measurements on the same child. All analyses were adjusted for baseline scores on the dependent variables. Missing values were not imputed. The data were analyzed using PASW (Predictive Analytics SoftWare; version 18.0.3).

## Results

**Figure 1:**
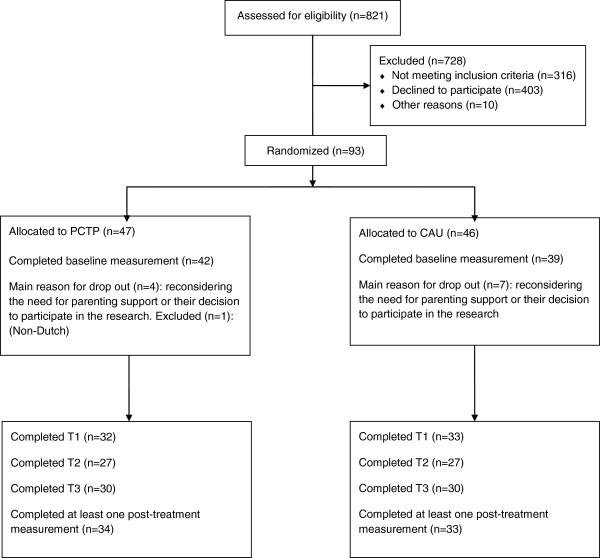
Flowchart of participants through the study, following CONSORT guidelines.

Figure 1 shows the flow of subject selection for the 2-year inclusion period [32]. Initially, 93 parents and their children were randomized to either PCTP (n = 47) or UC (n = 46). Eight parents dropped out immediately after randomization, reconsidering their need for parenting support or their decision to participate in the research, and did not provide a baseline measurement. Furthermore, three individual families did not complete the baseline measurement for various reasons, and were excluded from the analysis, as was one family who could not read or speak Dutch sufficiently, which should already have been excluded. Data on baseline measurements and at least one post-treatment measurement were available for 67 participants. In the ITT analysis, 81 participants with a baseline measurement were included. The results are for all participants with at least one post-intervention measurement (n = 67).

### Baseline

The PCTP and UC groups did not differ in terms of any of the background variables. The between-group differences were not statistically significant except for family size; the number of people per household was larger in the intervention group (*P* < 0.05). Prior to intervention, there were no significant differences between the study groups for any of the outcome variables, indicating that the randomization process had resulted in two similar groups (Table 1).

**Table 1:** Demographic and outcome measures of participants at baseline by treatment group (n = 67)

	**PCTP**	**UC**	**Total**	***P *** **value**
n	34	33	67	
Parent(s)				
Age of mother, mean ± SD	41.6 ± 4.92	40.94 ± 3.63	41.34 ± 4.34	NS
Age of father, mean ± SD	44.06 ± 5.47	42.48 ± 3.75	43.32 ± 4.77	NS
Dutch, %	96%	96%	96%	NS
Mother’s education (medium to high), %	58.3%	68.8%	63.3%	NS
Father’s education (medium to high), %	66.6%	67.7%	67.2%	NS
Both parents employed (>12 hours/week), %	57.6%	67.7%	62.5%	NS
Child				
Age, mean ± SD	10.57 ± 0.73	10.60 ± 0.65	10.59 ± 0.68	NS
Gender (boys), %	44.4%	67.6%	55.7%	
Household				
Single parent, %	25.0%	6.2%	16.2%	NS
Financial concerns, %	8.6%	15.6%	11.9%	NS
Family size (number of members), mean ± SD	4.34 ± 1.09	3.83 ± 0.78	4.07 ± 0.97	S
Baseline measures, mean ± SD				
SDQ-TDS	13.89 ± 4.42	13.03 ± 4.57	13.46 ± 4.48	NS
ECBI total score	104.17 ± 22.29	99.88 ± 19.42	102.06 ± 20.89	NS
PS mean score	3.34 ± 0.64	3.15 ± 0.56	3.25 ± 0.61	NS
PSI total score	28.50 ± 12.06	24.71 ± 7.66	26.66 ± 10.27	NS
PSBC total score	216.26 ± 42.45	230.27 ± 32.35	223.06 ± 38.26	NS
DASS total score	19.11 ± 22.57	12.82 ± 11.39	16.06 ± 18.18	NS

*Abbreviations*: DASS, Depression Anxiety Stress Scale; ECBI, Eyberg Child Behavior Inventory; NS, not significant; PCTP, Primary Care Triple P; PS, Parenting Scale; PSBC, Problem Setting and Behaviour Checklist; PSI, Parental Stress Index; SDQ-TDS, Strengths and Difficulties Questionnaire – Total Difficulties Score; UC, usual care.

### Effects on primary and secondary outcomes

**Figure 2:**
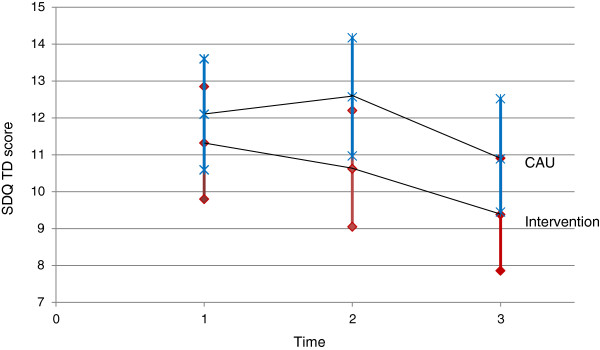
**Estimated means and 95% confidence intervals for the Strengths and Difficulties Questionnaire Total Difficulties Score (SDQ-TDS; primary outcome) by treatment group.** The analysis was corrected for baseline values. Intervention group is represented by the red line and diamonds for the point estimate and the 95% confidence interval borders. Usual care group is represented by the blue line and crosses for the point estimate and the 95% confidence interval borders.

For the primary outcome (SDQ-TDS) the difference between the PCTP and UC group after completion of the intervention was 0.77 (95% CI −1.37 to 2.91), after adjustment for baseline scores, which was not significant (*P* = 0.47). The difference increased to a maximum of 1.94 (95% CI = −0.30 to 4.19; *P* = 0.09), but remained non-significant at the 6-month follow-up, and decreased slightly (1.59, 95% CI = −0.56 to 3.76; *P* = 0.14) at the 12-month follow-up (Figure 2). A maximum difference of 5.81 (95% CI = −3.37 to 14.99; *P* = 0.21) was found on the ECBI at the 6-month follow-up. The highest mean improvement scores were also found for the SDQ and the ECBI at the 6-month follow-up.

No significant differences were detected between the treatment groups for the secondary outcomes (Table 2).

**Table 2:** Effects of Primary Care Triple P compared with UC: means at baseline, improvements from baseline, regression coefficients (B), and 95% CI expressing differences in outcomes after treatment adjusted for baseline, based on multi-level models (n = 67)

**Outcome**	**Group**	**Improvement**								
		**T0 to T1 (immediately after treatment)**			**T0 to T2 (6 months after treatment)**			**T0 to T3 (12 months after treatment)**		
		**Mean (SD)**	**B**	**95% CI**	**Mean (SD)**	**B**	**95% CI**	**Mean (SD)**	**B**	**95% CI**
SDQ^a^ (α = 0.60)	UC	0.96 ± 4.34	0.77	−1.37 to 2.91	0.96 ± 5.44	1.94	−0.30 to 4.19	2.29 ± 5.96	1.59	−0.56 to 3.76
	I	2.74 ± 4.04			3.57 ± 4.93			4.19 ± 5.0		
ECBI^a^ (α = 0.87)	UC	1.81 ± 13.48	4.99	−3.63 to 13.64	2.74 ± 13.62	5.81	−3.37 to 14.99	9.22 ± 17.71	5.18	−3.59 to 13.97
	I	8.53 ± 22.07			10.67 ± 19.24			15.29 ± 24.35		
PS^b^ (α = 0.84)	UC	0.05 ± 0.40	0.13	−0.04 to 0.31	0.04 ± 0.37	0.02	−0.17 to 0.21	0.02 ± 0.49	0.10	−0.07 to 0.29
	I	0.26 ± 0.37			0.15 ± 0.35			0.20 ± 0.46		
PSI^b^ (α = 0.88)	UC	1.21 ± 6.00	2.75	−0.87 to 6.37	0.66 ± 6.97	−0.51	−4.41 to 3.38	−1.35 ± 9.59	2.18	−1.51 to 5.89
	I	4.93 ± 9.09			1.10 ± 5.49			1.96 ± 8.71		
PSBC^b^ (α = 0.97)	UC	−0.20 ± 1.71	−0.54	−1.20 to 0.10	0.28 ± 0.90	−0.04	−0.71 to 0.62	0.31 ± 0.92	−0.39	−1.05 to 0.26
	I	0.82 ± 1.42			0.55 ± 1.67			0.84 ± 1.47		
DASS^b^ (α = 0.94)	UC	3.21 ± 10.33	−5.80	−12.28 to 0.67	3.55 ± 14.09	−5.38	−12.25 to 1.48	1.03 ± 14.60	−3.64	−10.26 to 2.96
	I	2.00 ± 24.43			3.86 ± 19.03)			2.83 ± 18.44		

*Abbreviations*: DASS, Depression Anxiety and Stress Scale; ECBI, Eyberg Child Behaviour Inventory; I, iIntervention; PCTP, Primary Care Triple P; M, Mean; SD, standard deviation; PS, Parenting Scale; PSBC, Problem Setting and Behaviour Checklist; PSI, Parenting Stress Index; SDQ, Strengths and Difficulties Questionnaire; UC, Usual care.

^a^Primary outcome.

^b^Secondary outcome.

In both treatment groups, psychosocial problems (SDQ, ECBI) and parenting stress (PSI) decreased significantly (*P* < 0.05). More detailed analyses of SDQ and ECBI syndrome scales identified a significant advantage of the PCTP arm for treatment of SDQ conduct problems (*P* < 0.05) at 6 and 12 months post-treatment (not shown). The ITT analyses, performed on all 81 participants, did not identify any significant differences for any of the primary or secondary outcomes, or for the SDQ conduct problems.

### Treatment integrity

The number of PCTP sessions varied from one to four. The intervention was discontinued before the entire intervention (that is, four sessions) had been completed if parents indicated that the support had been sufficient. In the UC group, nine parents indicated that they received parenting support, varying from advice to a house visit. Five parents and their child in the PCTP group received additional support in the period between baseline and first follow-up. Per-protocol analyses on those receiving at least two treatment sessions yielded similar findings as shown in Table 2.

## Discussion

This study evaluated the effects of PCTP on child problem behaviour, parenting behaviour, and parenting stress compared with UC provided by PCH. The study enrolled parents of children with mild psychosocial problems according to the SDQ. We found no significant differences between PCTP and UC on either the primary or secondary outcome measures, but PCTP yielded slightly better results than UC on most of these outcomes. Only in one SDQ field, namely, conduct problems, was a significant difference detected, which was in favor of the PCTP condition. In general, a decrease in child psychosocial problems and parenting stress was found for both PCTP and UC.

We found no significant advantages of PCTP on either primary or secondary outcomes. This contrasts with the findings for several previous studies on the effectiveness of PCTP, which suggested greater and more significant effects of this intervention [16,17,33] in terms of child and parent outcomes. There are several possible explanations for these differences. First, we compared the intervention with another treatment, UC provided by CHPs, whereas previous studies mostly compared a treated group with a group on a waiting list for such treatment [17,33]. The improvements in both treatment groups in our study during treatment support this explanation. Second, parents were included after an initial population-based screening to identify psychosocial problems in their child, whereas previous studies included only parents who explicitly requested advice about child behavioural problems or parenting issues [17,33]. This might have resulted in a different study population in terms of child age, characteristics of the participating parents, and nature of the detected problems. Third, the instruments used to assess our study outcomes were applied independently of the instruments used to monitor progression in parenting and child behaviour during and after the intervention. This might have affected the way parents completed the questionnaires. Fourth, we were unable to obtain the number of participants needed according to our power analysis, and small treatment groups may have led to the absence of a significant clinical effect of PCTP. However, our power analysis was based on a difference in improvement on the SDQ of three points as being clinically relevant [22], whereas we found a difference of only 1.94. Because this study is underpowered the precision of the estimate of effect is reduced, implying that the real effect could be bigger or smaller. Nevertheless, even if we had reached the intended sample size, it is unlikely that we would have found a substantial difference. Therefore, the advantage of PCTP seems limited compared with UC. Smaller effects may still have a relatively large effect on population health, given the large share of children with mild psychosocial problems [34]; however, it is doubtful whether such small effects outweigh the effort made per child by the parents, child, and professionals as involved. A fifth explanation could be that because some interventions were discontinued prematurely, they may have been less effective. PCTP is an protocol-based intervention, and treatment adherence is very important, thus deviations in its execution would have an influence on its effectiveness [35]. However, in our study the majority of the PCTP interventions were completed.

### Strengths and limitations

This study has some important strengths. First, we randomized to prevent selection and allocation bias, resulting in two comparable groups. Furthermore, the study evaluated the effectiveness of PCTP in a preventive healthcare organization delivered by regular staff from multiple centers, and therefore mirrors everyday practice. Contrary to earlier studies, we also assessed the long-term effects at 6 and 12 months after intervention in both the intervention and control group. A broad array of outcome measures gave an understanding of possible intervention effects in many areas of child behaviour and parenting. Moreover, to overcome any social desirability bias, parents did not complete the questionnaires in the presence of the CHP who conducted the intervention. Instruments to study the effect were offered to the parents separately from the treatment process.

This study also has some limitations. As already indicated, it was underpowered and the treatment integrity (that is, the number of delivered intervention sessions) was not optimal. During the trial, PCTP was implemented as routine care in some of the participating regions. To prevent contamination, we had to exclude these regions. Moreover, in the remaining regions, the inflow of eligible parents of children with mild psychosocial problems was lower than expected, because some parents were not invited to participate because of a high workload for CHPs or the reluctance of either the professional or the parent to participate in an RCT. This led to a lower than intended sample size. Participation of only a small proportion of eligible parents in this study may have affected the external validity of this study, that is, the application of the results of the trial to the general population of screening-detected parents and children. Nevertheless, this study reflects everyday practice in care. Furthermore, we only collected data on parent-reported child behaviour and parenting behaviour, and not from other information sources such as teachers or professionals. However, self-report has shown to be a good indicator for child problems [36].

## Conclusions

This study found no significant advantages of PCTP compared with UC for the outcomes measured. Therefore, evidence concerning the treatment superiority of PCTP is still not conclusive. However, because PCTP seems to reduce child psychosocial problems, particularly conduct problems, and to have no serious adverse effects, its implementation may still be justified, although it does not necessarily surpass UC in effectiveness. Because this study was underpowered, the conclusions need to be interpreted carefully. Further research in larger samples is needed to confirm our findings, particularly in the long term. Furthermore, research is needed to determine whether the costs of the large-scale implementation in PCH counterbalance its benefits [37].

## Abbreviations

CHP: Child healthcare professional; CONSORT: Consolidated Standards of Reporting Trials; DASS: Depression Anxiety Stress Scale; ECBI: Eyberg Child Behaviour Inventory; ITT: Intention-to-treat; PCH: Preventive Child Health Care; PCTP: Primary Care Triple P; PS: Parenting Scale; PSBC: Problem Setting and Behaviour Checklist; PSI: Parental Stress Index; SDQ-TDS: Strengths and Difficulties Questionnaire – Total Difficulties Score; Triple P: Positive Parenting Programme.

## Competing interests

The authors declare that they have no competing interests.

## Authors’ contributions

SAR and WS had the original idea for the project, wrote the study proposal, and obtained funding for the study. WS and DEMCJ wrote the study protocol, which was discussed by all authors leading to the final design. WS wrote the final manuscript, which was discussed, edited and revised by all authors. All authors contributed to the revisions, and read and approved the final manuscript.

## Pre-publication history

The pre-publication history for this paper can be accessed here:

http://www.biomedcentral.com/1741-7015/11/240/prepub
